# Effects of warning systems on the reactions of male and female drivers: A systematic review

**Published:** 2019-08

**Authors:** Mirbahador Yazdani, Mahdi Rezaei, Mohammad Saadati, Mahsa Jafari

**Affiliations:** ^*a*^Road Traffic Injury Research Center, Tabriz University of Medical Sciences, Tabriz, Iran.; ^*b*^School of Civil Engineering, Iran University of Science & Technology, Iran.

## Abstract

**Background:**

Warning systems are widely used as a safety option in cars. The aim of this study was to systematically investigate the reactions of male and female drivers after receiving the warnings.

**Methods:**

A literature search was done using Science Direct, Scopus and PubMed databases. The included articles have been published in 2003-2018. Articles were first screened reviewing their titles in order to exclude non-relevant articles. Then, the abstracts and full texts of the retained articles were reviewed. A manual search of journals was also performed.

**Results:**

Of 915 retrieved articles, 29 articles have been included in this review. In the included articles, 11 articles use forward collision warning (FCW) system, 6 articles use lane departure warning (LDW) system and 21 articles use other types of warning systems including intersection collision warning system, red light running collision warning, adaptive cruise control, etc. Most of the studies showed a significant difference between genders in the case of reaction-type variables. The variables that are significant in more studies are warning rate, crash rate and brake reaction time.

**Conclusions:**

Most of the included articles indicate that there are significant differences between male and female drivers after receiving warnings. This can be considered for customers to be more vigilant about choosing the appropriate warning system.

**Keywords:**

Systematic review, Gender, Vehicle, Warning, ADAS

